# Simultaneous Realization of Anomalous Reflection and Transmission at Two Frequencies using Bi-functional Metasurfaces

**DOI:** 10.1038/s41598-018-20315-2

**Published:** 2018-01-30

**Authors:** Xi Wang, Jun Ding, Bowen Zheng, Sensong An, Guohua Zhai, Hualiang Zhang

**Affiliations:** 1 Shanghai Key Laboratory of Multidimensional Information Processing, East China Normal University, Shanghai, 200241 China; 2 ECE Department, University of Massachusetts Lowell, Lowell, MA 01854 USA; 3 State Key Laboratory of Millimeter Waves, Southeast University, Nanjing, 210096 China

## Abstract

The capability to manipulating electromagnetic (EM) waves at the sub-wavelength scale has been enabled by metamaterials and their two-dimensional counterparts, metasurfaces. Especially, integrating two or more diverse functionalities into a single metasurface-based device is of great significance to meet the stringent requirements imposed by today’s high frequency components and systems. Here, we present a dual-band bi-functional metasurface structure that could simultaneously achieve anomalous reflection and transmission at two terahertz (THz) frequencies, respectively, under linearly-polarized incident waves. To demonstrate the property of the proposed metasurface, a number of dual-band bi-functional metasurface-based components that could tailor the reflected and transmitted waves simultaneously are designed and verified numerically. Moreover, it is shown that both the amplitude and phase responses of the reflected and transmitted waves at two operating frequency bands (wavelengths) can be manipulated using the proposed metasurface, providing a new and convenient way to construct multi-functional metasurfaces and corresponding electromagnetic devices.

## Introduction

Metasurfaces are a class of two-dimensional artificial materials with the ability of guiding the scattering waves due to the induction of abrupt phase change at the interface. They are made of properly arranged subwavelength scatterers, offering a new paradigm for designing ultrathin planar devices with the advantages of low profile and easy fabrication^1,2^. As artificial materials, metasurfaces possess the capabilities of controlling the phase, amplitude, and polarization of the incident EM waves, which do not exist in the nature and thus have enabled plentiful exotic applications^3–10^. Recently, significant progresses have been made on metasurfaces with multi-bands^11–17^, high-efficiency^18–23^, and polarization conversion^24–26^ using one or more layers from microwave to optical frequency regimes. Furthermore, from the perspective of high frequency systems, the combination of multiple functions in a single metasurface-based device is highly desired as it will lead to highly integrated systems with significantly reduced cost and size. Examples include a bi-functional metasurface (reflection-type or transmission-type), which can realize two functionalities (e.g. focusing and deflection) under different polarizations^27^; a three-layer metasurface producing broadband anomalous reflection or transmission under the forward/backward-propagating circularly-polarized incidence^28^; and a 1-bit coding metasurface composed of two resonators with the ability of splitting the input signal to two or four obliquely reflective beams according to different coding sequences at distinct frequencies^29^. It is noted that all the existing multifunctional metasurfaces operate in either transmission or reflection mode. To push the performance of metasurfaces and achieve more possible applications, it is meaningful to explore metasurface designs which can simultaneously manipulate transmitted and reflected EM waves at will.

In the design of metasurfaces, V-shaped resonators were firstly proposed and demonstrated to realize anomalous reflection and refraction phenomena by imparting the phase discontinuities and increasing new degrees of freedom^30^. Since then, it is widely applied as a basic building block to achieve a variety of functional components^31–34^. Meanwhile, the C-shaped ring resonator(CSRR) was presented and experimentally validated at the terahertz frequency band, which features the capability to tailor both the phase and amplitude responses simultaneously^12,13,35,36^, and became a popular metasurface structure (especially in the terahertz band). The complementary C-shaped slot resonator was also proved to have similar properties such as possessing the 2π continuous phase coverage and manipulating the phase and amplitude independently^37^.

In this work, a dual-band bi-functional metasurface cell (building block) operating at the terahertz band is proposed and designed, which is composed of two thin layers of meta-atoms separated by a polyimide substrate. When illuminated by a linearly-polarized terahertz wave, the proposed metasurface cell can operate in the reflection and transmission (refraction) modes at the higher and the lower frequencies, respectively. While working in the reflection mode, the top-layer meta-atoms function as the scatterers and the bottom-layer meta-atoms can be treated as a defected electric conducting surface to enhance the reflection efficiency. Similarly, the bottom-layer meta-atoms will dominate the transmitted wave at the lower frequency, at which the top-layer meta-atoms become almost transparent. Moreover, under each working mode (i.e. reflection/transmission mode), both phase and amplitude responses can be simultaneously tailored by the proposed metasurface building block. An elliptical hole is introduced at the bottom layer to reduce the interference between the two metasurface layers and can be used to further finely tune the amplitude and phase responses. Due to the unique features of the proposed bi-functional metasurface, it can be utilized to realize multi-functional devices (e.g., deflectors and multi-focusing lenses) at two distinct frequencies (operating in different modes). To the best of our knowledge, this is the first time that simultaneous control of both reflected and transmitted waves can be achieved using a single metasurface cell. It is expected that the proposed structure can be applied to realize various multi-functional or multi-band ultra-thin electromagnetic devices.

## Design and Results

### Metasurface working principle

**Figure 1:**
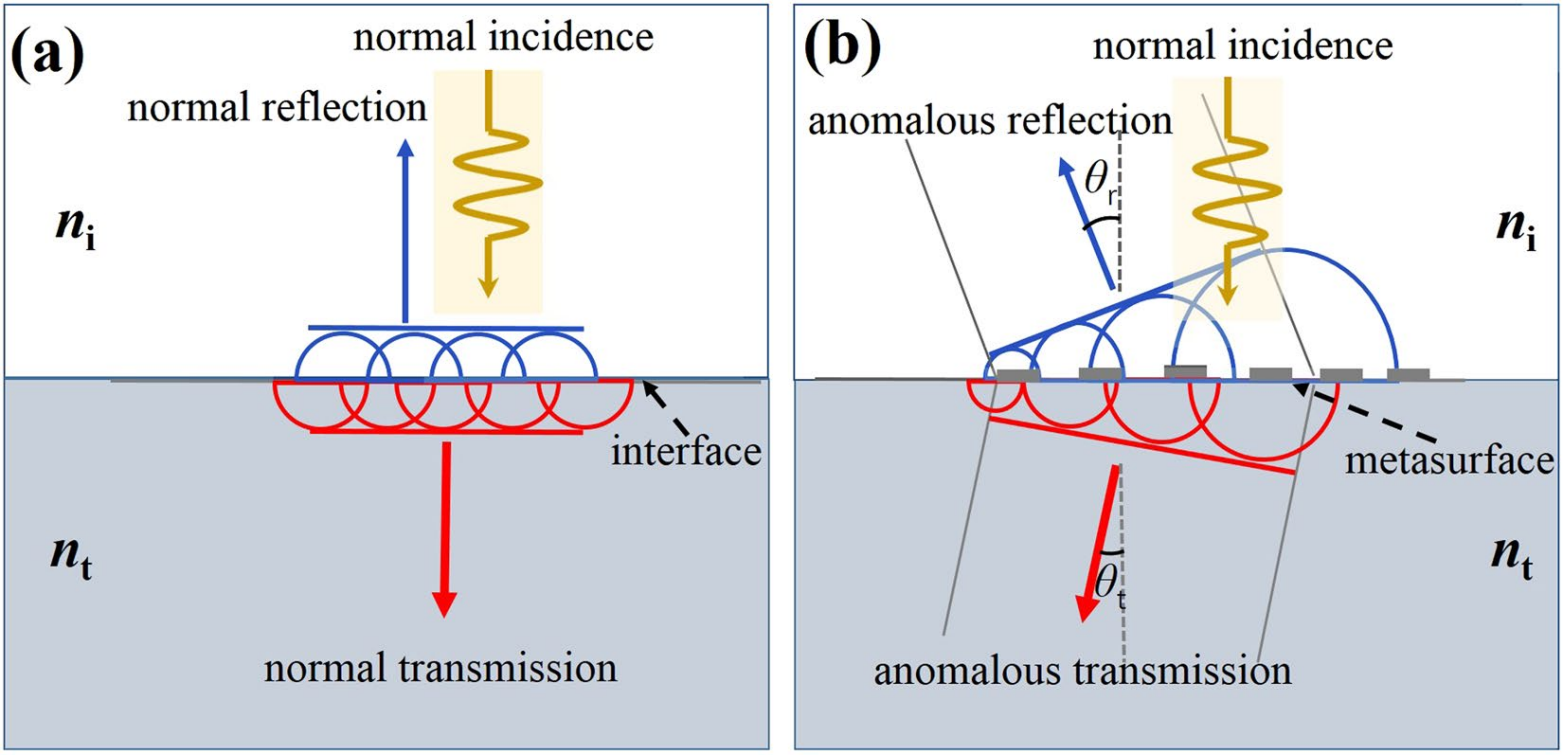
Schematic of the Huygens-Fresnel principle for deflections under a normal incidence with (**a**) a conventional interface (between two homogeneous media); (**b**) a metasurface interface.

In general, light propagation including reflection and refraction (transmission) can be interpreted by the Huygens-Fresnel principle in the view of the wave theory at the interface of two media^38^. Specifically, when illuminated by an EM wave, each point on the interface (as shown in Fig. 1(a)) can be treated as a secondary wave source to generate the new wavefront which governs the propagation properties of the incident wave. Under the case of no additional scatters introduced at the interface as shown in Fig. 1(a), with the normal incidence, the abrupt phase changes induced over the interface for both reflected and transmitted signals remain uniform. Therefore, the directions of both reflected and transmitted waves remain normal to the interface. In contrast, after applying a thin layer of metasurface to the interface (Fig. 1(b)), different abrupt phase changes can be induced by the metasurface cells at different locations. As a result, the reflected and transmitted wavefronts are no longer normal to the interface, which leads to the anomalous reflection and transmission as illustrated in Fig. 1(b).

In principle, the phase shift between adjacent scatterers (i.e. metasurface cells) controlling the angle of deflection (reflection and transmission) can be determined and calculated by the generalized Snell’s law^27^:

1 $$\sin ({\theta }_{r})-\,\sin ({\theta }_{i})=\frac{{\lambda }_{0}}{2\pi {n}_{i}}\frac{{\rm{d}}\phi }{{\rm{d}}x}$$

2 $${n}_{t}\,\sin ({\theta }_{t})-{n}_{i}\,\sin ({\theta }_{i})=\frac{{\lambda }_{0}}{2\pi }\frac{{\rm{d}}\phi }{{\rm{d}}x}$$

where *λ*_0_ is the free-space wavelength; *n*_t_ and *n*_i_ are the refractive indexes of the two media, *θ*_i_, *θ*_t_ and *θ*_r_ represent the angles of incidence, refraction and reflection, and *φ* represents the phase response. Correspondingly, the introduced metasurface cells could bring in arbitrary values of $\tfrac{{\rm{d}}\phi }{{\rm{d}}x}$ to generate the desired *θ*_t_ and *θ*_r_. For the applications of deflectors, in order to obtain the desired anomalous deflection angles, the value of $\tfrac{{\rm{d}}\phi }{{\rm{d}}x}$ is set as a constant, resulting in steady increasing or decreasing phases along the arrayed units. In practice, to realize any specific functions, each unit cell should be carefully designed so that the desired abrupt phase changes (as well as amplitude control which will be discussed later) can be achieved for both reflected and transmitted EM waves.

### Design of the Bi-functional metasurface cell

**Figure 2:**
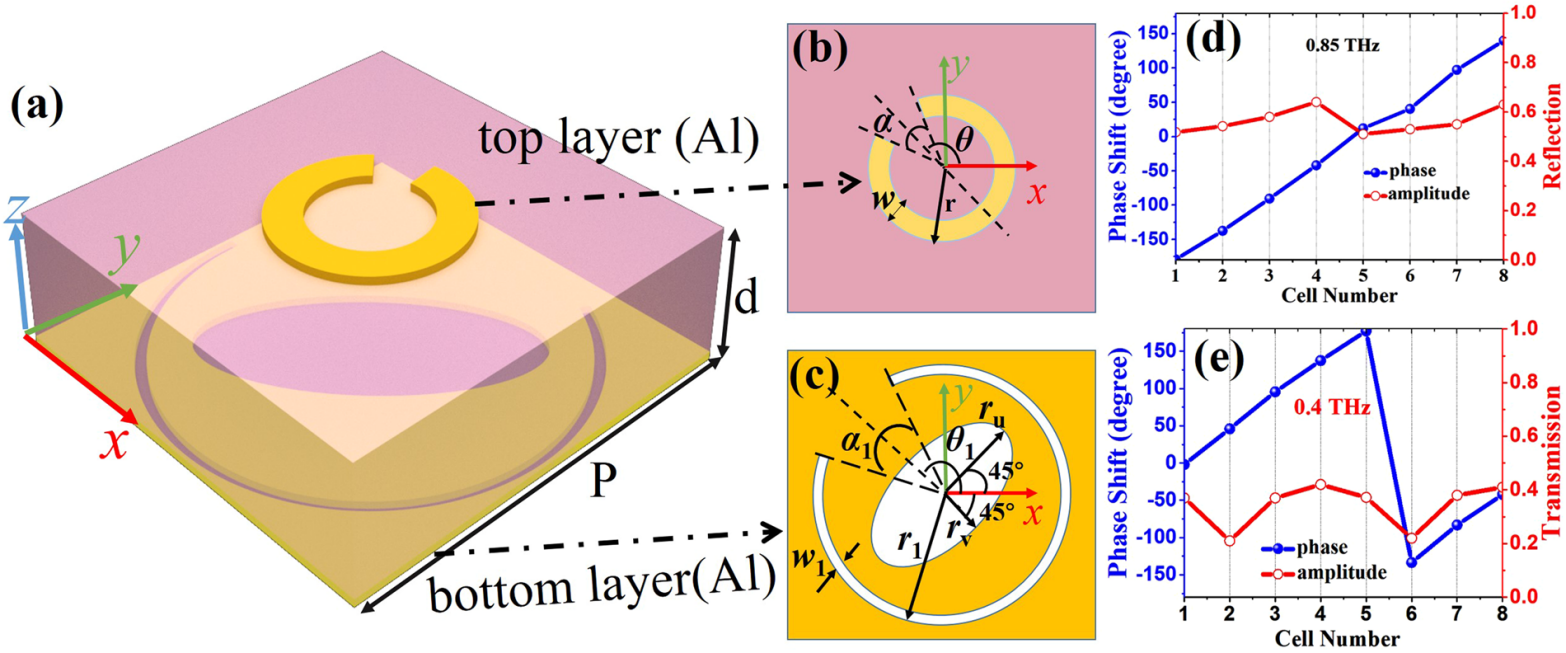
(**a**) Schematic of a building block of the bi-functional metasurface; (**b**) and (**c**) represent the top-layer and bottom-layer meta-atoms, respectively; (**d**) and (**e**) denote the cross-polarized phase and amplitude responses of eight metasurface cells with a gradient phase difference of 45° at 0.85 THz (reflection) and 0.4 THz (transmission), respectively, under the *x*-polarized incidence. (The parameters for these eight cells are detailed in Table 1).

A bi-functional metasurface cell is proposed to achieve the full 2π phase coverages for simultaneous reflection and transmission at two terahertz frequencies, respectively. The schematic of a unit cell is shown in Fig. 2(a), which is composed of a patterned layer (Fig. 2(b)) and a perforated layer (Fig. 2(c)) separated by a polyimide substrate (*ε*_r_ = 2.96, loss tangent *δ* = 0.012) with a thickness of *d* = 20 µm and a periodicity of *P* = 120 µm. The top (bottom) layer made of aluminum is designed to work at reflection (transmission) mode with the C-shaped (C-slot) resonator as the meta-atom because of its desired properties in the metasurface design. Without loss of generality, many other types of scatterers could also be utilized in the proposed structure. In order to reduce the mutual interference between different resonators, which is one of the critical issues in the design of multi-functional metasurfaces, an elliptical hole is introduced at the bottom layer as shown in Fig. 2(c), characterized by *r*_u_ and *r*_v_ and oriented with an angle of ±45° with respect to the *x*-axis. Moreover, the *r*_u_ and *r*_v_ can be adjusted to finely tune the phase and amplitude responses for both layers. As can be seen in Fig. 2(b) and (c), the outer radius, the opening angle, the width, and the orientation with respect to the *x*-axis of the C-shaped (C-slot) resonator are denoted as *r*(*r*_1_), *α*(*α*_1_), *w*(*w*_1_), and *θ*(*θ*_1_), respectively. In the following designs, the 2π phase coverage could be realized by mainly optimizing *r* (*r*_1_) and *α*(*α*_1_), and the amplitude response could be manipulated by rotating *θ*(*θ*_1_). The unit cell works in the cross-polarization mode and is simulated in CST Microwave Studio with periodic boundary conditions (more details could be found in the METHOD section). To realize the 2π phase coverage, eight metasurface cells are designed with a gradient phase difference of 45° at the two working frequencies. The optimized parameters are detailed in Table 1. It is known that an additional π phase difference could be achieved by changing the sign of *θ*(*θ*_1_) for the C-shaped (C-slot) resonator or flipping the resonators over the *x*-axis^27^, therefore, the later four metasurface cells (i.e., metasurface cells 5–8 in Table 1) can be obtained by flipping the first four (i.e., metasurface cells 1–4 in Table 1) over the *x*-axis. It is worth mentioning that the introduced elliptical hole has negligible effects on these important properties. The simulated phase and amplitude responses of the eight building blocks for the reflection (transmission) at *f*_1_ = 0.85 THz (*f*_2_ = 0.4 THz) are plotted in Fig. 2(d and e). Due to the cross-polarization working principle, the maximum cross-polarized reflection/transmission amplitudes of both the C-shaped and C-slot resonators could reach 0.5 in a homogeneous environment^39^. As illustrated in Fig. 2(d) and (e), the maximum transmission amplitude is around 0.38 at 0.4 THz, which is consistent with the case of a single CSRR with a transmission amplitude around 0.4^32^; while the reflection amplitude is around 0.6 at 0.85 THz, which is higher than 0.5 due to the introduced bottom layer acting as a defected ground plane. It is also shown in Fig. 2(d and e) that the 2π phase coverage can be achieved for reflection (transmission) at 0.85 THz (0.4 THz), thus, almost arbitrary reflected (transmitted) wavefronts can be generated with these eight building blocks.

**Table 1:** Detailed parameters of the designed metasurface cells.

Cell	#1	#2	#3	#4	#5	#6	#7	#8
*α*/*α*_1_(°)	29.5/115	36/143	42/55	63/95	29.5/115	36/143	42/55	63/95
*r*/*r*_1_(µm)	33/59	26.1/59.5	25.9/57.5	26.9/58.7	33/59	26.1/59.5	25.9/57.5	26.9/58.7
*θ*/*θ*_1_(°)	135/135	135/135	135/−135	135/−135	−135/−135	−135/−135	−135/135	−135/135
*r*_u_(µm)	46.5	47.5	47.5	43	21	23.1	25	31
*r*_v_(µm)	21	23.1	25	31	46.5	47.5	47.5	43

### Bi-functional deflectors

**Figure 3:**
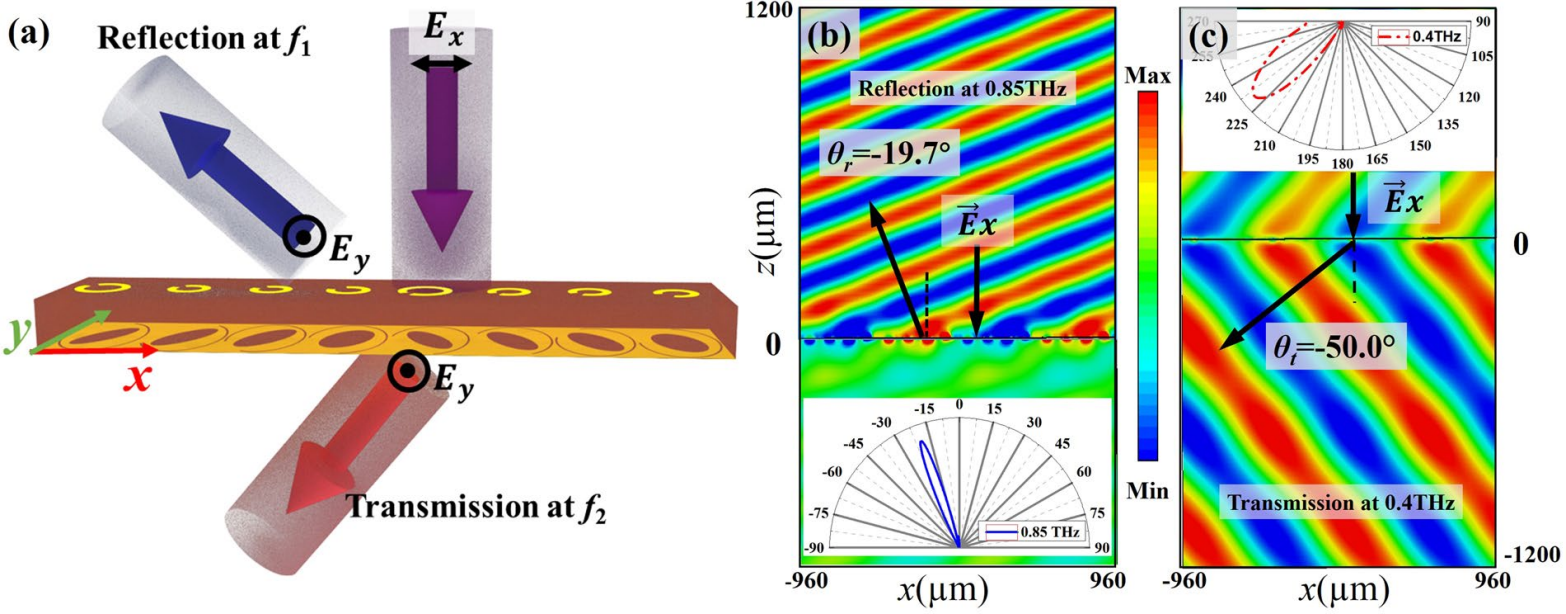
(**a**) Schematic of the proposed one-dimensional bi-functional deflector: the cross-polarized component of the normal incident wave is reflected at *f*_1_ and transmitted at *f*_2_. (**b**) and (**c**) *y*-polarized electric field distributions at 0.85 THz (reflection) and 0.4 THz (transmission) for the *x*-polarized normal incidence, respectively. (The far-filed responses are shown in the insets).

Beam deflectors are important optical elements which can be utilized to deflect the incident beams to the desired directions. Here, metasurface-based deflectors are presented to demonstrate simultaneous control of reflection and transmission of the proposed unit cell at two different frequencies. First, a simple one-dimensional (1D) dual-band bi-functional deflector is presented with the *x*-polarized incidence for both reflection and transmission at two frequencies, which is sketched in Fig. 3(a). A supercell is formed by the previously designed eight unit cells and repeated along both *x*- and *y*- directions. As can be seen in Fig. 3(a), with an *x*-polarized incident wave, the *y*-polarized reflected and refracted waves are detected at two frequencies (*f*_1_ and *f*_2_), which are mainly determined by the top-layer and bottom-layer meta-atoms, respectively. Figure 3(b and c) plots the simulated reflected (transmitted) *y*-polarized electric field of the 1D bi-functional deflector at 0.85 THz (0.4 THz) in XZ-plane. It can be seen that the normal incident wave at 0.85 THz (0.4 THz) is deflected to −19.7° (−50°), agreeing well with the theoretical value of −21.55° by Eq. (1) (−49.46° by Eq. (2)). The dual-mode deflecting functionality is further confirmed by the far-field responses illustrated in the insets of Fig. 3(b) and (c) for the reflection and transmission modes, respectively.

**Figure 4:**
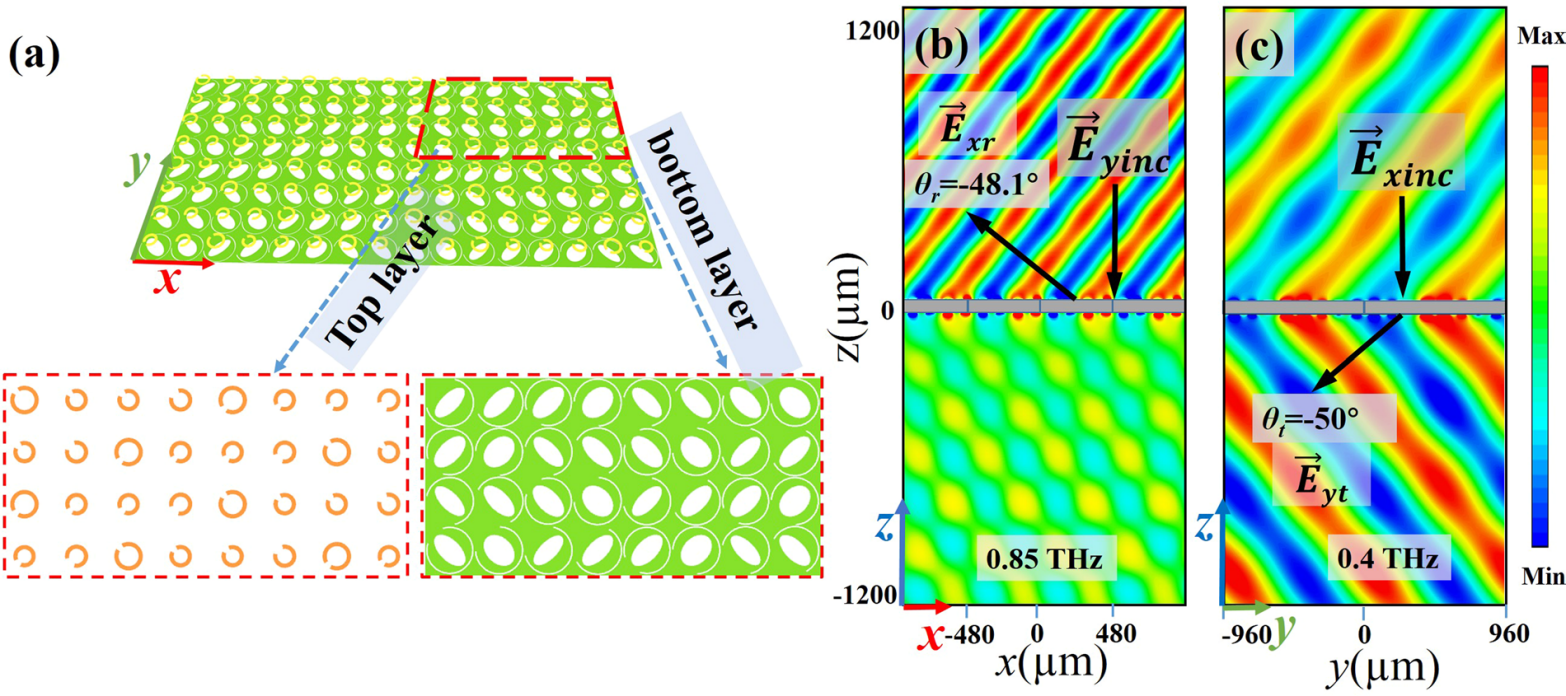
(**a**) Schematic of the proposed two-dimensional bi-functional deflector, composed of a top and bottom layers. (**b**) *x*-polarized electric field distributions at 0.85 THz (reflection) for the *y*-polarized normal incidence; (**c**) *y*-polarized electric field distributions at 0.4 THz (transmission) for the *x*-polarized normal incidence.

Next, a two-dimensional (2D) bi-functional deflector is designed with different polarized incident waves, which further demonstrates the independent wave control using the proposed structure at two frequencies. In this design, four top-layer meta-atoms chosen from aforementioned eight cells with a phase difference of 90° and the original eight bottom-layer meta-atoms are adopted to construct a supercell with 8 × 4 unit cells, as shown in the Fig. 4(a) with the red dotted line. Also displayed are the enlarged views of the top layer and the bottom layer of the supercell. The selected four top-layer (eight bottom-layer) meta-atoms are arranged along the *y*-axis (*x*-axis) with a phase difference of 90° (45°) and repeated along *x*-axis (*y*-axis), thus, the top (bottom) layer consists of eight identical columns (four identical rows). It is worth mentioning that the elliptical holes on the bottom layer are optimized to compensate for the slight phase fluctuation caused by the recombination of the top-layer and bottom-layer meta-atoms. Figure 4(b and c) plots the *x*-polarized (*y*-polarized) reflected (transmitted) electric wave at 0.85 THz (0.4 THz) in the XZ-plane (YZ-plane) under a normalized *y*-polarized (*x*-polarized) incidence. The cross-polarized deflected angle for the reflection (transmission) at 0.85 THz (0.4 THz) is −48.1° (−50°), agreeing well with the theoretical calculations.

### Bi-functional focusing lenses

**Figure 5:**
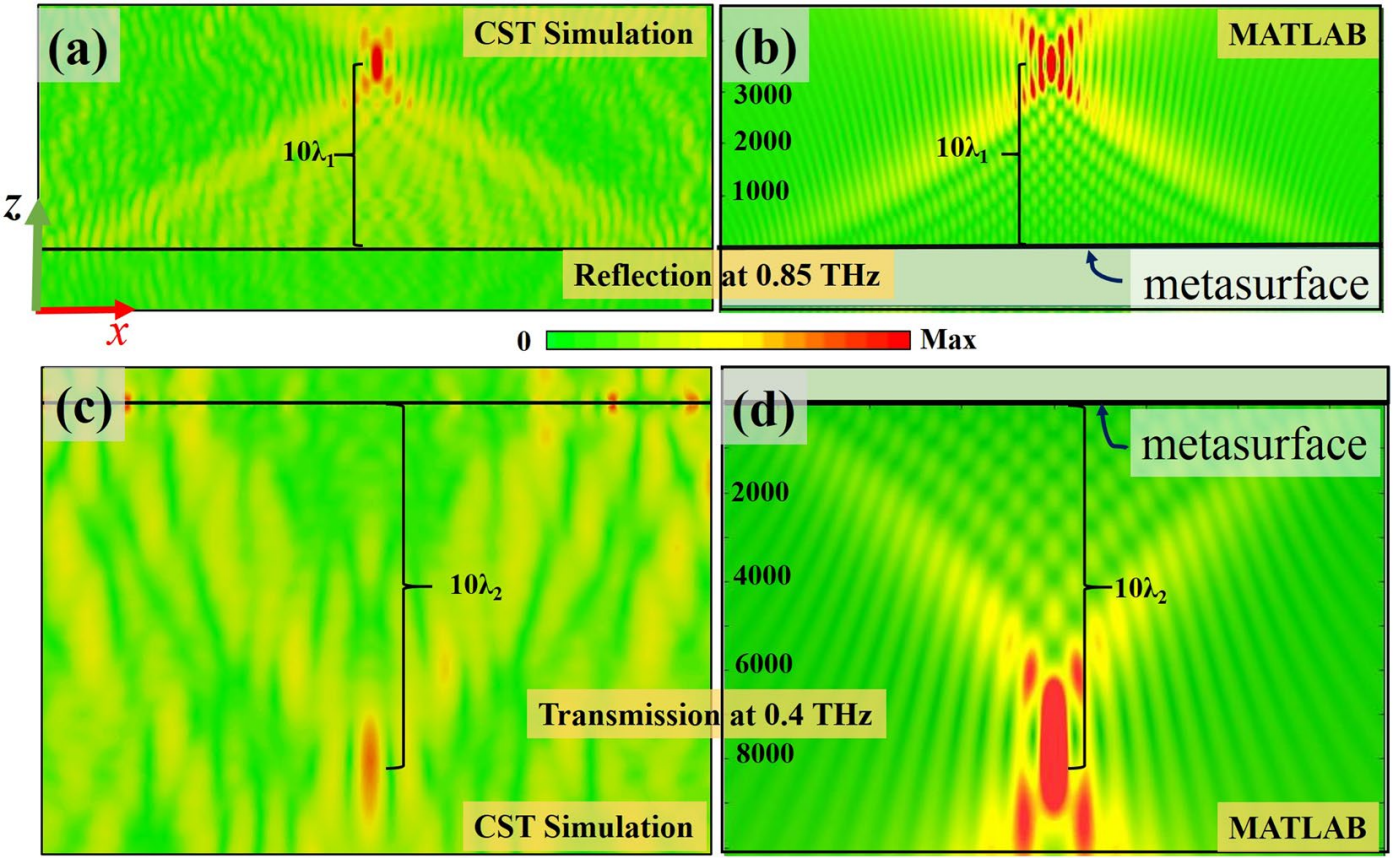
Intensity distributions of the *E*_*y*_ for the single-focal lens with (**a**) reflection at 0.85 THz and (**c**) transmission at 0.4 THz, respectively, under *x*-polarized normal incidence with F(*f*_i_) = 10c/*f*_i_. (λ_i_ = c/*f*_i_). (**b**) and (**d**) are the corresponding plots for (**a**) and (**c**) by theoretical calculation with MATLAB, respectively.

In addition to realizing the metasurface-based deflectors with the constant phase difference between adjacent unit cells, arbitrary phase profiles could be achieved with the proposed metasurfaces, which could be utilized to realize more complex devices/systems such as focusing lenses as presented in the following section. It is well-known that traditional lenses are grinded to specified shapes to satisfy the required phase profile through the propagation length difference, which makes them bulky and expensive. Due to the advance of nano-fabrication techniques and the emergence of metasurfaces, the required phase profile of a lens could be realized by the subwavelength metasurface unit cells, leading to planar lenses with both size and cost reductions. Here, a metasurface-based single-focal lens with dual focusing capabilities (reflection and transmission) at two different frequencies is demonstrated, using pre-described phase distributions. The cells are arranged along the *x*-direction. To realize the desired focusing effect, the phase profile *φ*(*x*) at the position *x* should follow the hyperbolic function as described below,

3 $$\phi (x,{f}_{i})={k}_{i}(\sqrt{{\rm{F}}{({f}_{i})}^{2}+{x}^{2}}-{\rm{F}}({f}_{i}))\quad i=1,2$$

where *k*_i_ = 2πc/*f*_i_ (c is the light speed) and F(*f*_i_) represent the free-space wavenumber and the focal length at frequency *f*_i_, respectively. In this design, F(*f*_i_) is set to be 10c/*f*_i_. The required phase distributions as calculated by Eq. (3) are discretized and realized by the previously designed eight metasurface building blocks at two frequencies. Note that the sizes of elliptical hole on the bottom layer are also modified to maintain the amplitude/phase responses at each frequency. The designed bi-functional single-focal lens consists of 101 cells along the *x*-direction (uniform along the *y*-direction). Its performance is shown in Fig. 5, which clearly demonstrates the desired dual-focusing behaviors in both reflection (Fig. 5(a)) and transmission (Fig. 5(c)) modes. Furthermore, we theoretically calculate the *E*_y_ field distribution in the XZ plane with MATLAB by modeling each meta-atom as a dipole with corresponding phase and amplitude distributions obtained by Eq. (3). The intensity distributions of the *E*_y_ for the single-focal lens under the reflection and transmission modes are plotted in Fig. 5(b) and (d), respectively. By comparing the results between the numerical simulations and the theoretical calculations, it can be concluded that the two sets of results agree well with each other (both of them are also consistent with the design goals).

**Figure 6:**
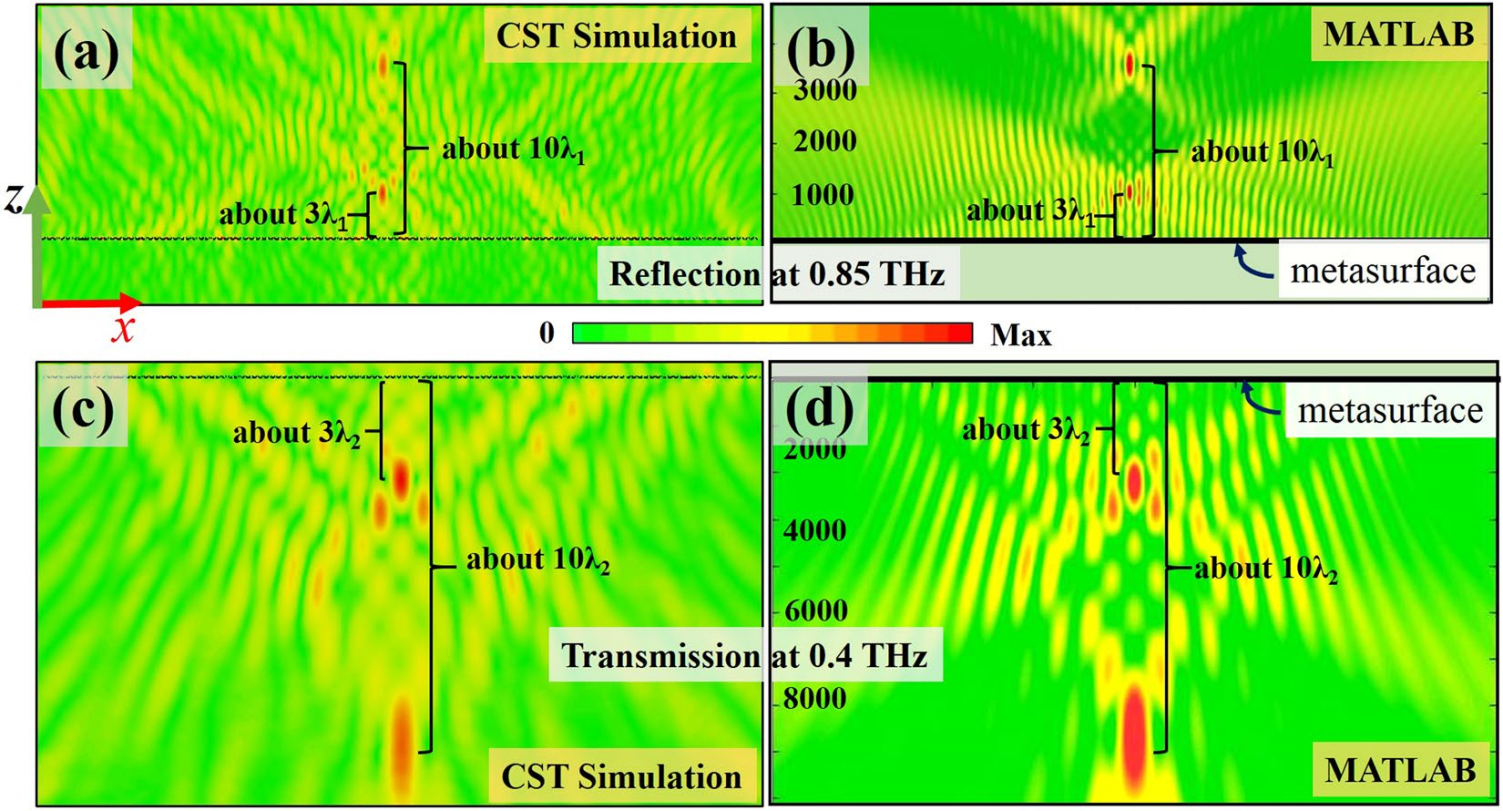
Intensity distributions of the *E*_*y*_ for the bi-foci lens with (**a**) reflection at 0.85 THz and (**c**) transmission at 0.4 THz by numerical simulation with CST, respectively, under *x*-polarized normal incidence with *a*_1_ = *a*_2_, F_1_(*f*_i_) = 3c/*f*_i_ and F_2_(*f*_i_) = 10c/*f*_i_. (**b**) and (**d**) are the corresponding plots for (**a**) and (**c**) by theoretical calculation with MATLAB, respectively.

Moreover, to demonstrate the capability of the proposed bi-functional metasurface for simultaneous amplitude and phase controls, a bi-foci lens at two different THz frequencies is presented. Different from the single-focal lens where only phase modulation is needed, to achieve a bi-foci lens with two focal spots F_1_(*f*_i_) and F_2_(*f*_i_), both the phase and amplitude responses of each cell (locating at different positions) need to be regulated accordingly. The phase and amplitude profiles *φ*(*x*) and *A*(*x*) at position *x* for a bi-foci lens follow the equation^37^:

4 $$A(x,{f}_{i}){e}^{-j\phi (x,{f}_{i})}={a}_{i1}{e}^{-j{k}_{i}(\sqrt{{{\rm{F}}}_{{\rm{1}}}{({f}_{i})}^{2}+{x}^{2}}-{{\rm{F}}}_{{\rm{1}}}({f}_{i}))}+{a}_{i2}{e}^{-j{k}_{i}(\sqrt{{{\rm{F}}}_{2}{({f}_{i})}^{2}+{x}^{2}}-{{\rm{F}}}_{{\rm{2}}}({f}_{i}))}\quad i=1,2$$

where *a*_1_ and *a*_2_ represent the amlpitudes at the two focal points and are set to be 0.5 at both frequencies (note: the single-focal lens presented in Fig. 5 can be treated as a special case with *a*_1_ = 0 and *a*_2_ = 1). In the designed dual-mode bi-foci lens, the two focal points are choosed as F_1_(*f*_i_) = 3c/*f*_i_ and F_2_(*f*_i_) = 10c/*f*_i_ at each frequency *f*_*i*_. As discussed before, the amplitude responses can be manipulated by rotating the oriention angle *θ* and *θ*_1_ of the top-layer and bottom-layer meta-atoms when illuminated with a linearly-polarized EM wave, resulting in the reflection/transmission amplitude modulations of the proposed structure. Although the variation of *θ* (*θ*_1_) of the top-layer (bottom-layer) meta-atom has a slight impact on the other one, the elliptical hole at the bottom layer can be adjusted to achieve the desired amplitude and phase responses at two frequencies for each unit cell. The simulated performance of the designed dual-mode bi-foci lens composed of 121 cells is illustrated in Fig. 6(a) and (c). Moreover, the theoretical calculations with MATLAB are plotted in Fig. 6(b) and (d). As can be seen in Fig. 6(a and d), the reflective (transmissive) bi-foci phenomenon can be observed at 0.85 THz (0.4 THz) with almost the same intensity at the two designed focal spots, agreeing well with the design goal (*a*_1_ = *a*_2_). It is noteworthy that dual-mode multi-foci lenses can be also realized by adding more terms in Eq. (4).

## Conclusions

In summary, a novel bi-functional metasurface building block has been proposed to manipulate reflected and transmitted waves at two different THz frequencies, respectively, consisting of a top-layer and a bottom-layer meta-atoms separated by a polyimide spacer. Both amplitude and phase manipulations could be achieved by the proposed metasurface unit cell operating in both the reflection mode (at the higher frequency) and the transmission mode (at the lower frequency) simultaneously, which could be of great importance in many applications. Specifically, the reflective and transmissive responses of the unit cell are dominated by two different types of meta-atoms on the top-layer and bottom-layer, respectively. The critical issue of mutual interference between the two layers is addressed by introducing an additional elliptical hole on the bottom layer, offering more degrees of freedom to tailor the amplitude/phase responses at the two working frequencies. Based on the proposed metasurface cells, several dual-band bi-functional electromagnetic devices including two deflectors (1D and 2D) and two focusing lenses (single-focus and bi-foci) are designed and numerically verified at two arbitrarily selected THz frequencies, the ratio of which could be varied from 1.3 to 3 or even larger. The simulated results agree well with the theoretical calculations and design goals. It is expected that the designed metasurfaces could pave the way towards achieving multi-functional metasurface-based devices, impacting various high-frequency systems for applications such as tele-communications and imaging.

## Methods

### Simulations

All simulations are performed by the commercial software Computer Simulation Technology (CST) Microwave Studio. In the simulation of the metasurface unit cells, unit cell boundary conditions are set along both the *x* and *y* directions and open boundary condition (add space) is applied in the *z* direction. For the simulations of the deflectors, periodic boundary conditions are set in both *x* and *y* directions for the supercells. For the simulations of the lenses, periodic boundary condition is set in *y* direction and open boundary condition (add space) is applied in the *x* direction.
